# Health surveillance of deployed military personnel occasionally leads to unexpected findings

**DOI:** 10.1186/1741-7015-10-126

**Published:** 2012-10-24

**Authors:** Alexander C McFarlane

**Affiliations:** 1 Centre for Traumatic Stress Studies, University of Adelaide, Adelaide SA 5000, Australia

**Keywords:** PTSD, inflammation, physical illness, cytokines, allostatic load, sensitization, combat, military

## Abstract

Post-traumatic stress disorder (PTSD) can be caused by life threatening illness, such as cancer and coronary events. The study by Forbes *et al. *made the unexpected finding that military personnel evacuation with medical illness have similar rates of PTSD to those evacuated with combat injuries. It may be that the illness acts as a nonspecific stressor that interacts with combat exposures to increase the risk of PTSD. Conversely, the inflammatory consequence of systemic illness may augment the effects to traumatic stress and facilitate the immunological abnormalities that are now being associated with PTSD and depression. The impact of the stress on cytokine systems and their role in the onset of PTSD demands further investigation. Military personnel evacuated due to physical illness require similar screening and monitoring for the risk of PTSD to those injured who are already known to be at high risk.

## Background

Health surveillance of deployed military personnel yields a wealth of information, occasionally revealing findings that were not anticipated. The high rates of post-traumatic stress disorder (PTSD) found by Forbes *et al. *[1] in personnel medically evacuated from Iran and Afghanistan while on deployment is surprising. Those military personnel who were medically ill had similar rates of PTSD as those evacuated for injuries sustained in combat. Little information is provided about these illnesses, with the most common being a gastrointestinal disorder (45.7%), limiting the conclusions that can be reached. However, there has been a debate in the literature about the extent to which physical illnesses can act as the stressor leading to the onset of PTSD, such as with cardiac disease and cancer [2,3]. The results of this study cast interesting new light on this question about the interrelationship between PTSD and illness in a setting where there are already high levels of exposure to traumatic stress.

### Stress, inflammation and psychiatric disorders

This paper does not address the question as to whether the increased rate of PTSD arose as a consequence of the sense of perceived threat from these illnesses or whether the PTSD arose from the combat exposures that these individuals had suffered prior to becoming medically ill. The medically ill group was over-represented in those who had significant traumatic deployment exposures. One possible mechanism to explain this increased rate could be that the individual reactions to that deployment experience has been modified by the subsequent sense of vulnerability associated with the physical illness that led to them being deployed home. In essence, an individual who becomes physically unwell is less resilient psychologically in the aftermath of combat exposures.

An alternative explanation may be that the individuals who are systemically unwell are more at risk of PTSD due to the biological activation of inflammatory and other neurobiological systems that underpin the neurobiological dysregulation associated with PTSD [4,5]. For example, increased cytokines have been observed in PTSD and inflammation is increasingly being considered as an aetiological mechanism in psychiatric diseases [6].

These increased rates of disorder are not limited to PTSD but are also observed for other common mental disorders, and argue that the underlying process is likely to be a shared vulnerability to all disorders. This conceptual framework is consistent with the model of allostatic load, which argues that a range of disorders are associated with dysregulation of a series of homeostatic mechanisms and that this represents a common vulnerability to both physical and mental disorders [7]. Physical and psychological stress activate similar systems that increase the risk of a range of disorders [8].

A further possibility is that PTSD and psychological distress were risk factors for gastrointestinal disorders and the other illnesses that led to their medical evacuation. While PTSD and depression are risk factors for cardiovascular events [9], an intriguing possibility is that those with these disorders were also at greater risk of other disorders as a consequence of changed immune reactivity.

The increased rates of PTSD in those with physical injuries are consistent with the published literature. However, the same inflammatory mechanisms may contribute to the outcome of this group where the consequences of pain and the physiological load associated with injury play a role in the onset and course of PTSD.

Finally, it is important to state that the mean time elapsed since deployment was 2.0 years (IQR 0.8 to 4.5 years). The individuals who are unwell at the time of the study may not reflect those who had been significantly symptomatic at an earlier time. As populations are followed after injury, evidence shows that there is a group who has coped well in the immediate aftermath of the injury but progressively developed an increase of symptoms leading to delayed onset PTSD. Sensitisation and kindling play a critical role in the aftermath of the exposure to traumatic events [10]. Individuals who have sustained either a physical injury or illness may be at particular risk of this trajectory because of their likely comorbidities.

## Conclusions

In the military setting, those who are medically evacuated either because of illness or injury are readily identifiable and hence, being populations at risk, should be systematically followed in clinical settings as a group at significant risk of comorbid psychiatric injury. Simple strategies can be put in place for screening in medical environments where they require ongoing care if the risk is anticipated. In these settings, PTSD frequently goes undetected despite the significant benefits that can be gained by effective treatment as demonstrated in a screened population who attended emergency departments after the London terrorist bombings [11].

Past wars have provided information on the marked attrition of military manpower due to losses from infectious disease, climate extremes and not just battle injuries. These have led to the origins of military medicine. Military psychiatry emerged to curtail manpower losses from mental disorders. Ironically, this study highlights that those who suffer from combat stress are not the only group who deserve the attention of the psychiatrist in the longer term. Those who become physically ill are also clearly a group at risk.

## Abbreviations

IQR: interquartile range; PTSD: Post-traumatic stress disorder.

## Competing interests

I am a member of the specialist reserves of the RAAF. I have received funding from the Australian Defence Force to conduct the 2010 ADF Mental Health Prevalence and Wellbeing Study and studies of Australian Veterans of the Middle East Area of Operations. I have not been directed in anyway, but the Australian Defence Force has influenced the content of this commentary.

## Author information

AC McFarlane is currently the Head of the University of Adelaide Centre for Traumatic Stress Studies and also the Senior Adviser in Psychiatry to the Australian Defence Force and the Australian Centre for Posttraumatic Mental Health. He is an international expert in the field of PTSD and the impact of disasters.

## Pre-publication history

The pre-publication history for this paper can be accessed here:

http://www.biomedcentral.com/1741-7015/10/126/prepub

## References

[B1] Forbes HJ Jones N Woodhead C Greenberg N Harrison K White S Wessely S Fear NT What are the effects of having an illness or injury whilst deployed on post deployment mental health? A population based record linkage study of UK Army personnel who have served in Iraq or Afghanistan BMC Psychiatry 2012 12 178 10.1186/1471-244X-12-178 23095133 PMC3507752

[B2] Kangas M Henry JL Bryant RA Posttraumatic stress disorder following cancer. A conceptual and empirical review Clin Psychol Rev 2002 22 499 524 10.1016/S0272-7358(01)00118-0 12094509

[B3] Shemesh E Yehuda R Milo O Dinur I Rudnick A Vered Z Cotter G Posttraumatic stress, nonadherence, and adverse outcome in survivors of a myocardial infarction Psychosom Med 2004 66 521 526 10.1097/01.psy.0000126199.05189.86 15272097

[B4] Baker DG Nievergelt CM O'Connor DT Biomarkers of PTSD: neuropeptides and immune signaling Neuropharmacology 2012 62 663 673 10.1016/j.neuropharm.2011.02.027 21392516

[B5] Bob P Raboch J Maes M Susta M Pavlat J Jasova D Vevera J Uhrova J Benakova H Zima T Depression, traumatic stress and interleukin-6 J Affect Disord 2010 120 231 234 10.1016/j.jad.2009.03.017 19359044

[B6] Loftis JM Huckans M Morasco BJ Neuroimmune mechanisms of cytokine-induced depression: current theories and novel treatment strategies Neurobiol Dis 2010 37 519 533 10.1016/j.nbd.2009.11.015 19944762 PMC2995293

[B7] McEwen BS Gianaros PJ Central role of the brain in stress and adaptation: links to socioeconomic status, health, and disease Ann N Y Acad Sci 2010 1186 190 222 10.1111/j.1749-6632.2009.05331.x 20201874 PMC2864527

[B8] Dunn AJ Effects of cytokines and infections on brain neurochemistry Clin Neurosci Res 2006 6 52 68 10.1016/j.cnr.2006.04.002 18079991 PMC2136407

[B9] Kubzansky LD Koenen KC Spiro A Vokonas PS Sparrow D Prospective study of posttraumatic stress disorder symptoms and coronary heart disease in the Normative Aging Study Arch Gen Psychiatry 2007 64 109 116 10.1001/archpsyc.64.1.109 17199060

[B10] McFarlane AC The long-term costs of traumatic stress: intertwined physical and psychological consequences World Psychiatry 2010 9 3 10 20148146 10.1002/j.2051-5545.2010.tb00254.x PMC2816923

[B11] Brewin CR Fuchkan N Huntley Z Scragg P Diagnostic accuracy of the Trauma Screening Questionnaire after the 2005 London bombings J Trauma Stress 2010 23 393 398 20564372 10.1002/jts.20529

